# 4EGI-1 induces apoptosis and enhances radiotherapy sensitivity in nasopharyngeal carcinoma cells via DR5 induction on 4E-BP1 dephosphorylation

**DOI:** 10.18632/oncotarget.7824

**Published:** 2016-03-01

**Authors:** Weiyuan Wang, Jiao Li, Qiuyuan Wen, Jiadi Luo, Shuzhou Chu, Lingjiao Chen, Zhenzhen Qing, Guiyuan Xie, Lina Xu, Mohannad Ma Alnemah, Meirong Li, Songqing Fan, Hongbo Zhang

**Affiliations:** ^1^ Department of Pathology, The Second Xiangya Hospital, Central South University, Changsha, Hunan, China; ^2^ Department of Oncology, The Second Xiangya Hospital, Central South University, Changsha, Hunan, China

**Keywords:** nasopharyngeal carcinoma, apoptosis, radiotherapy, 4EGI-1, DR5

## Abstract

The eIF4F complex regulated by a various group of eIF4E-binding proteins (4E-BPs) can initial the protein synthesis. Small molecule compound 4EGI-1, an inhibitor of the cap-dependent translation initiation through disturbing the interaction between eIF4E and eIF4G which are main elements of the eIF4E complex, has been reported to suppress cell proliferation by inducing apoptosis in many types of cancer. And death receptor 5 (DR5) is a major component in the extrinsic apoptotic pathway. However, the correlation among 4EGI-1, DR5 and 4E-BPs have not been discovered in NPC now. Therefore, we intend to find out the effect of 4EGI-1 on the apoptosis process of NPC and the relationship among 4EGI-1, DR5 and 4E-BPs. Our results revealed a significant down regulation of DR5 expression in NPC tissues, which inversely correlated with lymph node metastasis status and clinical stages. Depressed DR5 expression was an independent biomarker for poor prognosis in NPC, and elevated DR5 expression showed longer overall survival time in 174 NPC patients. Besides, 4EGI-1 induced apoptosis in NPC cells through the DR5-caspase-8 axis on 4E-BP1 and eIF4E dephosphorylation exerting positive influence on their anti-tumor activities. The induction of DR5 also sensitized NPC cells to radiotherapy, and the SER was 1.195. These results establish the death receptor pathway as a novel anticancer mechanism of eIF4E/eIF4G interaction inhibitor in NPC.

## INTRODUCTION

Nasopharyngeal carcinoma (NPC) is one of the common head and neck malignant tumors with the specificity of geographical distribution and ethnicity [1]. Radiotherapy or chemoradiotherapy remains the first choice of treatment for NPC patients. But those traditional methods are still not effective enough to prevent NPC from recurrence and metastasis [2–6]. Thus, identifying a comprehensive individualized treatment is a crucial issue for improving the conditions and increasing the survival rates for patients with advanced nasopharyngeal carcinoma.

The initial step of protein synthesis is the binding of the eukaryotic translation initiation factor 4E (eIF4E) to the 7-methylguanosine (m7-GpppG) 5′ cap of messenger RNAs. The eIF4E expression level is adjusted by the expression level of phosphorylation form of 4E-BPs. Unphosphorylated or hypophosphorylated 4E-BPs exhibit a high affinity to eIF4E and repress translation, on the contrary, hyperphosphorylated 4E-BPs lose their affinity with eIF4E and drive translation [7–9]. Around 30% numbers of cancers exhibit elevated eIF4E expression, which is associated with poor prognosis [10]. Overexpression of eIF4E induces cell proliferation by encoding some proliferation- and survival-promoting proteins (e.g., cyclins, c-myc, and Bcl-xL.) [11, 12]. Besides, some studies discovered dysregulated phosphorylation of 4E-BPs in cancers also linked to poor outcomes [10, 13]. Consequently, it is useful to design or improve drugs under pathological conditions in which eIF4E activity or global translation is upregulated [9, 14, 15].

Apoptosis is mainly regulated by the intrinsic pathway and extrinsic pathway according to the different startups [16, 17]. A variety of external factors, which act as an initiator, induce apoptosis through different signaling pathways. TRAIL - R1 (DR4) and TRAIL - R2 (DR5) are two members of death receptors (DRs), containing death structure domain and transmitting the cell death signals into cells. When tumor necrosis factor-related apoptosis-inducing ligand (TRAIL) integrates with DR4, or DR5, the adaptor Fas-associated protein with death domain (FADD) and caspase-8 will be further recruited, which lead to caspase-8 and APO-1/FAS signaling pathway activation [17]. So many anticancer drugs based on this apoptosis mechanism have an exact effect [18]. In addition, agonistic antibodies against DR4 and DR5 are on the stage of clinical test in phase I and II clinical trials and well tolerated [17, 19].

Small molecule compound 4EGI-1 (eIF4E/eIF4G interaction inhibitor) with the similar function to 4E-BPs could disrupt the eIF4E/eIF4G interaction and promote the combination of 4E-BP1 and eIF4E. This compound down-regulated tumor associated proteins against tumor cell growth without apparent toxicity [20]. Our previous study have found that 4EGI-1 increased the DR5 expression, decreased the c-FLIP expression and enhanced the pro-apoptotic effect of TRAIL in lung cancer as a single reagent or combined with TRAIL [21]. But the significance of apoptosis induction and radiosensitivity on the anti-tumor activities of eIF4E/eIF4G interaction inhibitor remains undefined in NPC. So we are interested in its therapeutic activity in nasopharyngeal cancer and its relationship with DR5 and 4E-BP1 now. Here, we detected expression of DR4 and DR5 proteins in NPC and non-cancerous nasopharyngeal epithelial tissues, results indicated that DR5 was inversely correlated with lymph node metastasis status, clinical stages. Subsequently, low DR5 expression might be an independent biomarker for poor prognosis in NPC. Besides, we proved that 4EGI-1 inhibited the expression level of phosphorylation form of 4E-BPs leading to activate the DR5-caspase8 axis, which resulted in apoptosis, growth inhibition and sensibilization of radiotherapy in NPC cells.

## RESULTS

### Association between expression of DR5 and DR4 proteins and clinicopathological features of NPC patients

**Figure 1:**
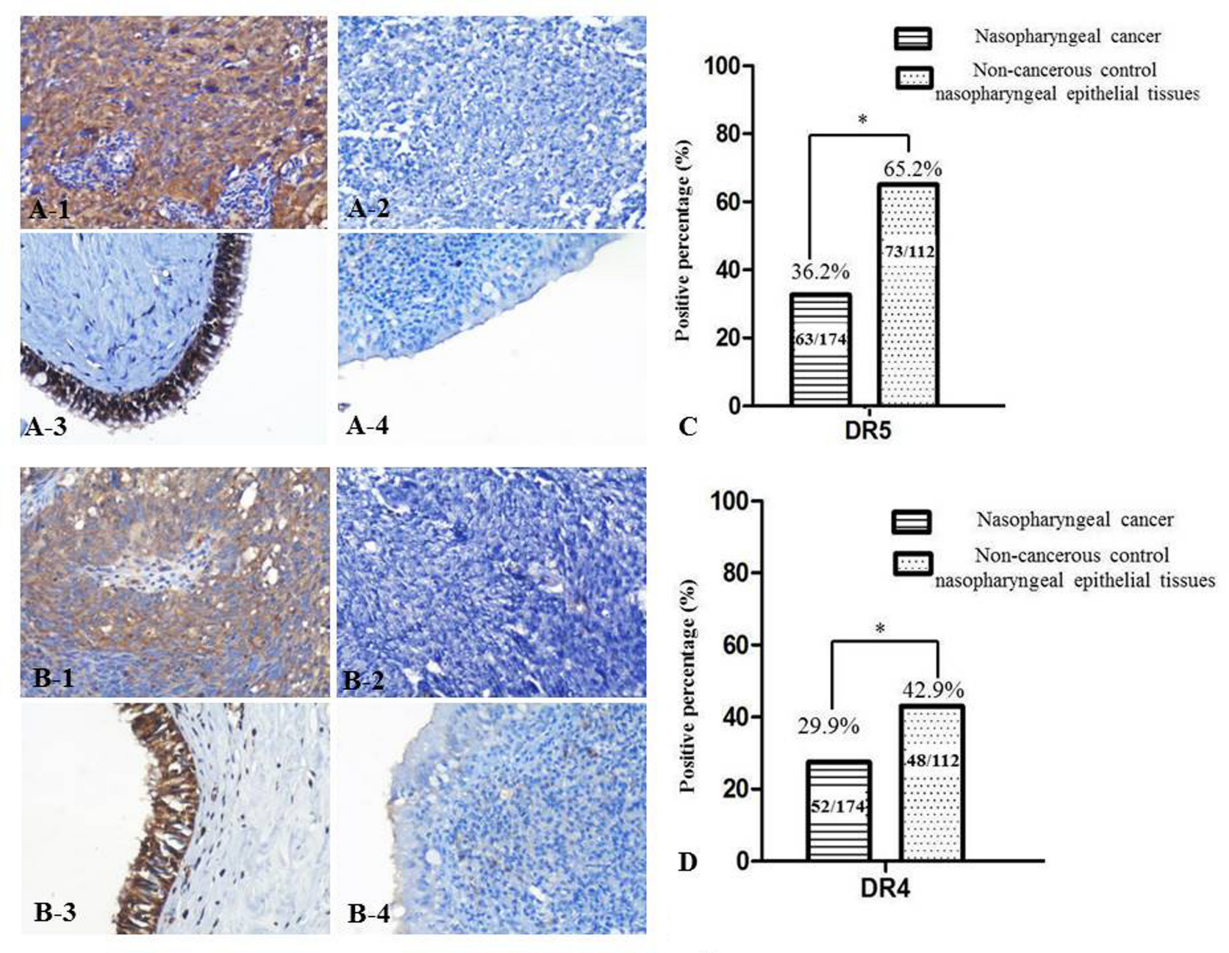
DR5 and DR4 expression decreased in NPC tissues Strong expression of DR5 protein was identified in NPC cells cytoplasm **A-1.** no expression of DR5 was identified in NPC tissues **A-2.** positive staining of DR5 was recognized in the non-cancerous control nasopharyngeal epithelia **A-3.** negative staining was showed in the control nasopharyngeal epithelia **A-4.** strong expression of DR4 protein was identified in NPC cells cytoplasm **B-1.** no expression of DR4 was showed in NPC tissues **B-2.** positive staining of DR4 was recognized in the control nasopharyngeal epithelia **B-3.** negative staining was indicated in the control nasopharyngeal epithelia **B-4.**, (20×, IHC, DAB staining). The percentage of positive expression of DR5 in non-cancerous control nasopharyngeal epithelial tissues was higher than that in NPC tissues **C.** The percentage of positive expression of DR4 in non-cancerous control nasopharyngeal epithelial tissues was higher than that in NPC tissues **D.**

Expression of DR4 and DR5 proteins in 174 cases of NPC and 112 cases of non-cancerous nasopharyngeal epithelia were detected by immunohistochemistry (IHC). Positive expression of DR5 was identified in the cytoplasm and nuclei of NPC cells (Figure 1-A1 and 1-A3), DR4 staining was mainly located in cells cytoplasm, a few cases were also stained in cytoplasm and nucleus (Figure 1-B1 and 1-B3). Positive DR5 and DR4 expression rates in NPC tissues (36.2%, 29.9%) were significantly lower than in non-cancerous control nasopharyngeal epithelial tissues (65.2%, 42.9%), (p<0.05) (Figure 1C, 1D).

We also analyzed association between expression of DR5 and DR4 and clinicopathological features of NPC patients including age, gender, histologic type, clinical stages, lymph node metastasis (LNM) status and overall survival rate by univariate χ2 test. Date shown in Table 1 indicated that the positive expression of DR5 in patients with LNM was lower than those without LNM (p=0.002); besides, NPC patients in the later stage (III and IV) showed lower DR5 expression than NPC patients in the early stage (I and II) (p=0.011). The results also showed a strongly positive correlation between positive expression of DR5 and the overall survival rate of NPC patients (P = 0.011). DR5 protein expression had no statistical difference with NPC patients' age, gender or histologic type (P > 0.05). In addition, no significant association was discovered between expression of DR4 and age, gender, histologic type, clinical stages, LNM status and overall survival rate of NPC patients (P > 0.05) (Table 1).

**Table 1:** Association between expression of DR5 and DR4 and clinicopathological features of NPC patients (n= 174)

Characteristics (n)	DR5			DR4		
	+ (%)	−(%)	P-value	+ (%)	−(%)	P-value
**Age (yr)**						
≤40 (n=59)	21(35.6)	38(64.4)		17(28.8)	42(71.2)	
>40 (n=115)	42(36.5)	73(63.5)	0.904	35(30.4)	80(69.6)	0.825
**Gender**						
Female (n=38)	13 (34.2)	25(65.8)		13(34.2)	25(65.8)	
Male (n=136)	50 (36.8)	86 (63.2)	0.772	39(28.7)	97(71.3)	0.510
**Histological type**						
DNC (n=14)	5(35.7)	9(64.3)		4(28.6)	10(71.4)	
UDNC (n=160)	58(36.3)	102(63.8)	0.968	48(30.0)	112(70.0)	0.911
**Clinical stages**						
Stages I-II (n=72)	34(47.2)	38(52.8)		21(29.2)	51(70.8)	
Stages III-IV (n=102)	29(28.4)	73(71.6)	0.011	31(30.4)	71(69.6)	0.862
**LN status**						
LNM (n=124)	36(29.0)	88(71.0)		37(29.8)	87(70.2)	
No LNM (n=50)	27(54.0)	23(46.0)	0.002	15(30.0)	35(70.0)	0.983
**Survival status**						
Alive (n=111)	49(44.1)	62(55.9)		36(32.4)	75(67.6)	
Dead (n=63)	14(22.2)	49(77.8)	0.004	16(25.4)	47(74.6)	0.330

Abbreviations: DNC: Differentiated non-keratinized nasopharyngeal carcinoma, UDNC: Undifferentiated non-keratinized nasopharyngeal carcinoma, LN: lymph node; LNM, lymph node metastasis.

### Impact of expressions of DR4 and DR5 on the prognosis of NPC patients

**Figure 2:**
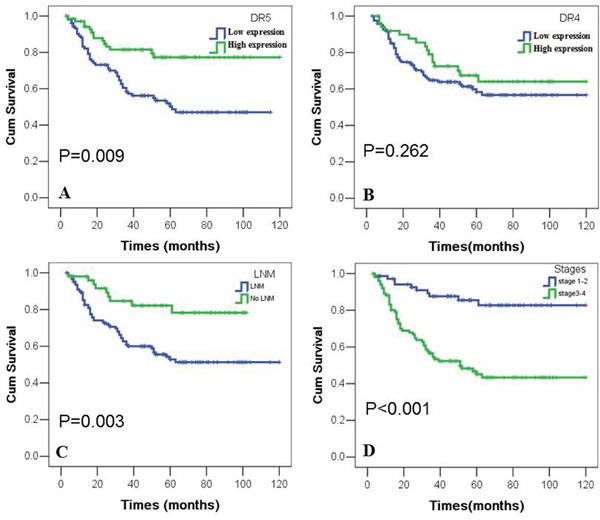
Kaplan-Meier overall survival curves of NPC patients with expression of DR4 and DR5 and clinicopathological features Kaplan-Meier analysis was used to plot the overall survival curves of 174 cases of NPC patients with different expression of DR4 and DR5, which statistical significance was assessed by log-rank test. NPC patients with lower expression of DR5, LNM or later stages showed worse overall survival rates compared to patients with higher DR5, no LNM and earlier stages (P=0.009, P=0.003, P<0.001, two sided, respectively). Positive expression of DR4 had no significantly correlation with overall survival rates of NPC patients (P>0.05, two sided).

Objective to further examine the correlation between the expression of DR4 or DR5 proteins and their importance for clinical prognosis of NPC patients, we used the Kaplan-Meier analysis to plot the survival curve of all 174 NPC patients, and statistical significance was assessed using the log-rank test. The overall survival rate for NPC patients with lower DR5 expression was significantly shorter than those with higher DR5 expression (p=0.09) (Figure 2A). In addition, NPC patients with LNM leaded to poor overall survival rate (p=0.03) (Figure 2C). And patients in earlier stages (I and II) had higher overall survival rate than those in advanced stages (III and IV) (p<0.001) (Figure 2D). The expression of DR4 had nothing to do with the prognosis of NPC patients (p>0.05) (Figure 2B).

The multivariate Cox's proportional hazard regression model was carried out to further evaluate whether DR4 and DR5 were independent prognostic parameters for NPC patients. As summarized in Table 2. Lower expression of DR5 protein could be used as an independent prognostic marker (p=0.021), as did clinical stages, LNM status and treatment strategy (p<0.01, p=0.004, p=0.008, respectively). But DR4 protein expression, histological type, age and gender had no significant correlation with the prognosis of NPC patients.

**Table 2:** Summary of multivariate analysis of Cox proportional hazard regression for overall survival in 174 cases of NPC patients

Parameter	Wald	SE	Sig.	Exp (B)	95.0% CI for Exp (B)	
					Lower	Upper
**DR5**	5.290	0.308	0.021*	0.493	0.270	0.901
**DR4**	1.531	0.292	0.216	0.697	0.393	1.235
**clinical stages**	14.948	0.350	0.000*	3.872	1.948	7.690
**LNM status**	8.211	0.370	0.004*	2.886	1.398	5.957
**Histological type**	1.934	0.414	0.164	1.779	0.790	4.009
**Treatment strategy**	7.141	0.267	0.008*	0.490	0.291	0.827
**Age**	0.111	0.286	0.739	1.100	0.628	1.925
**Gender**	0.047	0.329	0.829	1.107	0.563	2.047

Abbreviations: LNM, lymph node metastasis. *:*p*<0.05

### 4EGI-1 inhibits proliferation and induces apoptosis in human NPC cell lines

**Figure 3:**
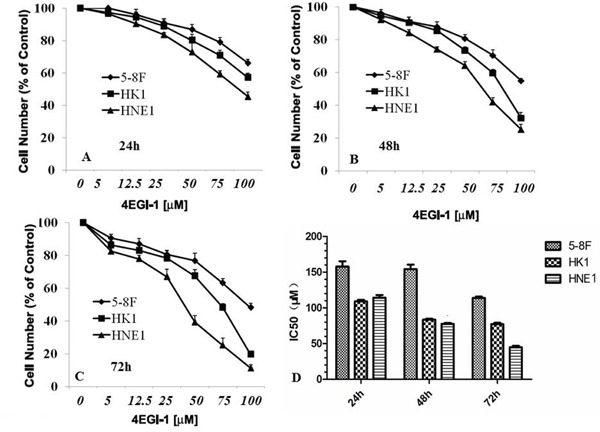
4EGI-1 inhibited the proliferation of NPC cell lines 5-8F, HK1 and HNE1 cell lines in 96-well plates were treated with serial concentrations of 4EGI-1 for 1 day **A.**, 2 days **B.**, or 3 days **C.** and their proliferation were measured by the SRB assay. The IC_50_ dosage of 4EGI-1 of 5-8F, HK1 and HNE1 cell lines for 24h, 48h or 72h were determined using the SRB assay **D.**

The sulforhodamine B assay (SRB) revealed the growth inhibition effect of a dilution series concentration of 4EGI-1 in all NPC cell lines including in our study. And the growth suppression of 4EGI-1 to NPC cells possessed obvious time dependence and dose dependence (Figure 3A, 3B, 3C and 3D). The sensitivity of 4EGI-1 was generally heterogeneous in different NPC cell lines, and IC50 values of various NPC cells treated 24h, 48h or 72h by 4EGI-1 were displayed respectively in Figure 3D. HNE1 cell line was the most sensitive one to 4EGI-1, and the IC50 of 4EGI-1 was about 50μM at 72h. Whereas 5-8F cell was the most insensitive to 4EGI-1 among all tested cell lines, and the IC50 of 4EGI-1 in 5-8F cell was about 120μM at 72h (Figure 3D). So, HNE1 cell line was used for the follow-up experiments research.

**Figure 4:**
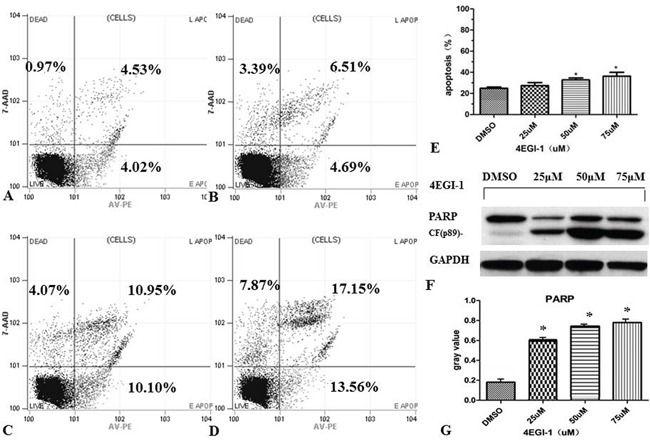
4EGI-1 augmented the apoptosis of NPC cell lines HNE1 cells were seeded in six-well plates. Apoptosis of HNE1 cells were detected by FCM 24 h after treating by DMSO **A.**, 25μm 4EGI-1 **B.**, 50μm 4EGI-1 **C.**, 75μm 4EGI-1 **D.**, individually. All experiments were repeated 3 times. Compared to the control, apoptosis rates increased in the 50μm and the 75μm groups, p<0.05 **E.** Detecting the expression of cleaved-PARP in incremental concentration of 4EGI-1 treated HNE1 cells, the expression of cleaved-PARP was upregulated in 4EGI-1 treated groups, GAPDH staining was used as loading control, p<0.05 **F-G.**

The flow cytometry (FACS) was used to further investigate whether 4EGI-1 exerts anti-cancer effect on NPC cells through inducing apoptosis. The experiment can be divided into four groups: vehicle control group (DMSO), small dose group (25μM), medium does group (50μM) and large dose group (75μM). We first exposed HNE1 cells to different doses of 4EGI-1 or DMSO as previously mentioned, and then stained them with Annexin-V-PE and PI to measure apoptosis rates after incubation for 24 hours (Figure 4A, 4B, 4C and 4D). FACS analysis showed that: in 50μM group and 75μM group, cells had more higher early apoptosis rates and total apoptosis rates than the vehicle control group (*P*<0.05); there were no statistical differences between 25μM group and vehicle control (*P*>0.05) (Figure 4E). Beyond that, western blotting analysis found the expression of cleaved-PARP was clearly upregulated in 4EGI-1 groups with the increasing concentration of 4EGI-1 (Figure 4F and 4G). These results demonstrated that appropriate doses of 4EGI-1 may suppress HNE1 cell proliferation through inducing cell apoptosis. About there was no obvious apoptosis under the light exposure of 4EGI-1(25μM group), it might be due to the condition that small dosage of 4EGI-1 could injury the DNA, but was not sufficient to cause cell state changes.

### 4EGI-1 induces apoptosis in NPC cell line via DR5 induction on 4E-BP1 dephosphorylation

**Figure 5:**
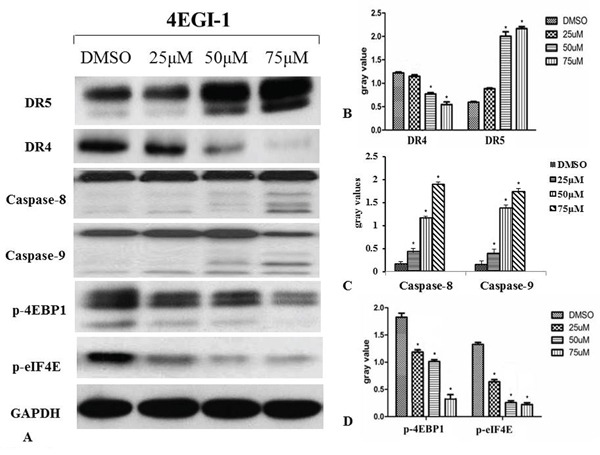
Western blotting was used to detect the activation of the death receptor pathway during apoptosis induced by 4EGI-1 in NPC cell lines Increased activity of the death receptor pathway in nasopharyngeal carcinoma cells was confirmed by treatment of eIF4E/eIF4G interaction inhibitor 4EGI-1. The inhibition of phosphorylation of 4EBP1 and eIF4E activated the DR5/caspase-8 axis in HNE1 cells, as demonstrated by inhibited expression of p-4EBP1 and p-eIF4E and enhanced expression of DR5, caspase-8 and caspase-9. But there was reduction in expression of DR4 compared to the vehicle control group. With the augment of concentration of 4EGI-1, the more obvious effect appeared. GAPDH staining was used as loading control. All data were representative of three independent experiments, p<0.05.

4EGI-1 induced classical apoptosis in HNE1 cells at high concentrations (50μM and 75μM). The apoptosis was preceded by induction of DR5 at concentration of 50μM group followed by cleavage of caspase-8 and −9 within 24h. Significant p-4EBP1 expression was inhibited in HNE1 cell line by 4EGI-1. More concerning, 4EGI-1 inhibited eIF4E phosphorylation much more rapidly and profoundly compared with 4EBP1 phosphorylation in the HNE1 cell line. The markers concerned before were affected by 4EGI-1 in a dose-dependent manner (Figure 5).

**Figure 6:**
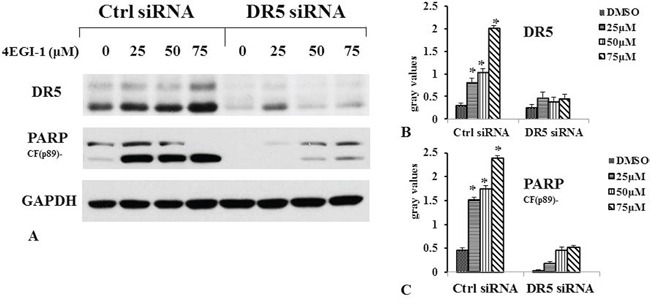
4EGI-1 induced apoptosis in NPC cell line via DR5 HNE1 cells were cultured in a six-well plate and, the next day, transfected with 60 nM control (Ctrl) or DR5 siRNA. Forty-eight hours after transfection, the cells were treated with 25, 50 or 75μM 4EGI-1 respectively and then harvested for detection of DR5 and cleaved PARP by western blot. Columns indicate means of duplicate experiments; bars, ±SE, p<0.05.

Furthermore, we determined whether elevated DR5 mediated enhancement of 4EGI-1-induced apoptosis by knocking down DR5 expression and then measuring its impact on 4EGI-1-induced apoptosis. Transfection of DR5 siRNA not only decreased the basal levels of DR5 but also reduced the 4EGI-1–induced DR5 up-regulation without dose-dependent, indicating successful knockdown of DR5 expression (Figure 6). Accordingly, the 4EGI-1 potently induced PARP cleavage in control siRNA–transfected cells but only minimally in DR5 siRNA-transfected cells in dose-dependent manner. Thus, these results indicated that 4EGI-1 induced apoptosis in NPC cells via DR5 on 4E-BP1 and eIF4E dephosphorylation, which could promote the combination of 4E-BP1 and eIF4E.

### 4EGI-1 sensitizes NPC cell line to radiation therapy through upregulating expression of DR5

**Figure 7:**
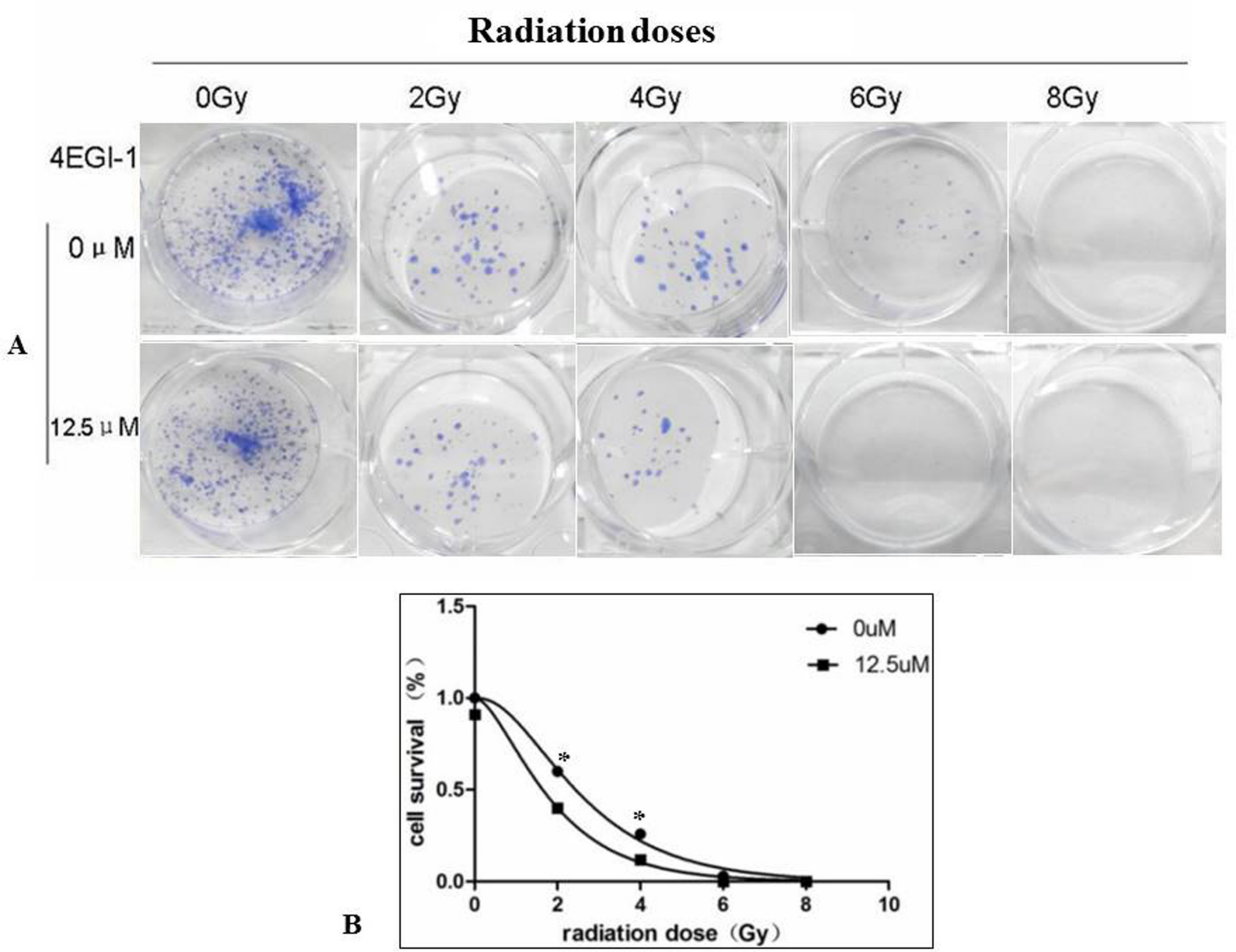
Combined treatment with 4EGI-1 and radiotherapy enhanced radiotherapy sensitivity in NPC cell lines Results of colony formation assays **A.** and survival curves **B.** for HNE1 cell line exposed to progressively higher doses of radiotherapy alone or combined with the 12.5μm of 4EGI-1 treatment, *p* < 0.05.

**Figure 8:**
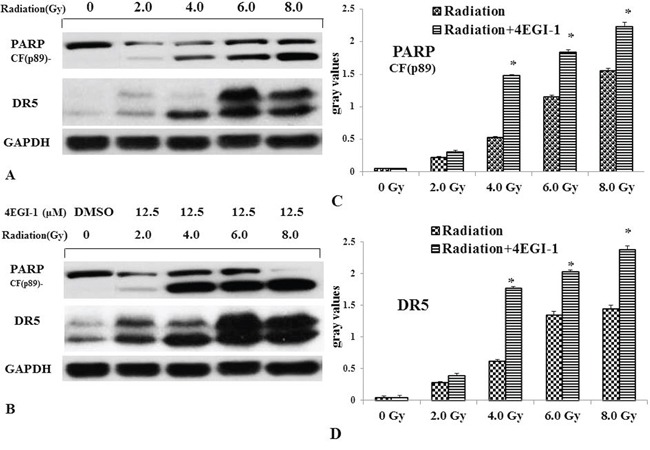
Validation of up-regulation of DR5 in NPC cells treated by radiotherapy alone or combined with 4EGI-1 treatment Lysates were prepared as described in methods. In HNE1 cell line DR5 and cleaved-PARP was analyzed in untreated cells (lane 1), 2Gy alone (lane 2), 4Gy alone (lane 3), 6Gy alone (lane 4) and 8Gy alone (lane 5) as basic parameters **A.** Before accepting previous radiotherapy, HNE1 cells were treated by 12.5μm of 4EGI-1. And then we detected the expression of DR5 and cleaved-PARP **B.** Every cell line shows a different cleavage pattern according to the rate of apoptosis induction. GAPDH staining was used as loading control. Compared to the radiotherapy alone, the expression of cleaved-PARP **C.** and DR5 **D.** increased in the combined treatment group. The mapped blots represent each one of three independently performed immunoblots.

Because the clinical outcome of NPC patients with a low DR5 expression level is worse than that of those with a high level of DR5 expression. Increasing DR5 in NPC cells might improve the outcome of radiation-based treatment. Luckily, the expression of DR5 could be improved by 4EGI-1. To further explore whether 4EGI-1 contributed to the radiosensitivity of HNE1 cells through upregulating DR5, cells were divided into 2 groups: the radiotherapy alone and the combined 4EGI-1 treatment group. According to the groups, the adherent cells were treated 24h with or without 4EGI-1, and then replaced the fresh complete medium and received irradiation at a single dose of 0, 2, 4, 6 or 8Gy respectively. The Graphpad Prism 5 software was used to calculate dose-survival curve and obtain relative values. Under the 2Gy dose radiation exposure, HNE1 cells had a greater inhibitory effect on colony formation and appeared significantly lower survival rates in the 4EGI-1 treatment group than the control ones (p<0.05); This phenomenon also existed at dose of 4Gy exposure (p<0.05). The sensitizing enhancement ratio (SER) was 1.195. However, there were no differences among other groups (P>0.05) (Figure 7A-7B). Besides, 4EGI-1 in combination with radiotherapy strongly induced activation of DR5 and enhanced cleaved PARP induction compared with radiotherapy alone by Western blotting analysis (Figure 8A–8D). With the augment of dose, the more obvious synergistic effect appeared. These data suggested that 4EGI-1 might contribute to the radiosensitivity of HNE1 cells through upregulating DR5 expression.

## DISCUSSION

In this study, we firstly verified that lower DR5 expression was a novel biomarker to predict poor prognosis of NPC patients. Accordingly, we wanted to look for some solutions. We proved that 4EGI-1 induced apoptosis and enhanced radiotherapy sensitivity in NPC cell line via DR5 induction on 4E-BP1 dephosphorylation. As far as we know, this is the first report to definitively prove that DR5 induction contributes significantly to the radiosensitivity of eIF4E/eIF4G interaction inhibitor such as 4EGI-1.

TRAIL is the member of the cytokine tumor necrosis factor (TNF) superfamily. TRAIL - R1 (DR4) and TRAIL - R2 (DR5) are two members of death receptors (DRs), containing death structure domain and transmitting the cell death signals into the cells. Here, we gave evidence that DR5 expression inversely correlated with LNM status and clinical stages; depressed DR5 expression was an independent biomarker for poor prognosis in NPC, and elevated DR5 expression showed longer overall survival time in 174 cases of NPC patients. However, there were no association between DR4 expression and clinicopathological features and the prognosis of NPC. The findings are consistent with others reported that wild-type TRAIL has higher affinity for DR5 than DR4, so the role of DR4 or DR5 is not the same in different tumors [27, 28]. Colon cancer patients with high DR4 expression had lower survival rate and easier relapse [22]. Inversely, DR4 did not affect the survival rates of patients with lung cancer, cervical cancer or ovarian cancer [23–25].

Emerging evidence supports targeting 4E-BP1 and eIF4E phosphorylation in cancer. Our findings discovered that exposure to gradient concentration of 4EGI-1 caused a profound loss of 4E-BP1 and eIF4E phosphorylation, both are likely required for the activation of DR5-caspase8 axis and subsequent killing of NPC cells, and 4EGI-1 enhanced the affinity of 4E-BP1 and eIF4E to bring down the translation efficiency of protein through repressing the phosphorylation of 4EBP1 and eIF4E. This consistent with our former and other findings [21, 26]. However, the possible mechanism by which DR5 is induced by 4EGI-1 could not rule out. DR5 transcription is regulated by p53 following DNA damage [27, 28] or CHOP after ER stress [29]. According to the researches, the expression of DR5 was mainly regulated by CHOP induced by ER stress when the phosphorylation of 4E-BP1 was abrogated by 4EGI-1 [21, 30]. It is tempting to speculate that the DR5 expression is elevated via induction of CHOP induced by ER stress in NPC. As for small dose of 4EGI-1 had no effect on the apoptosis of HNE1 cells, we hold that small dose of 4EGI-1 is able to impair the DNA and upregulate cleaved-PARP expression, but the damage could be repaired by cells. Interestingly, the expression of DR4 in HNE1 cell line treated by 4EGI-1 gradient concentration was opposite to the DR5 expression. This phenomenon was also reported by Bychkov and his colleague. They discovered the sensitivity of death receptors of cancer cells can be shifted from DR4 to DR5 by chemotherapeutic drug. So we reason that this theory might be applied to our study, but the exact mechanism remains to be further study.

Radiotherapy plays an irreplaceable role to cure NPC on account of the particularity of the anatomical position of NPC [31]. Whereas the study found that 5-year survival rate of NPC patients suffered radiotherapy alone was only 34-52%. Radiation biological effect is influenced by many factors, such as oxidative stress, glutathione, cell cycle and so on [32, 33]. But the correlation between elevated DR5 and radiosensitivity had not been reported. The results displayed: 4EGI-1 strongly synergize with radiotherapy to induced cancer cell killing through elevated DR5. The value of N, Do and Dq were lower in 12.5μM group than 0μM group, which meant cell repair ability dropping, cell damage threshold decreasing and radiation resistance reducing. And the SER which was calculated according to the value of Do and Dq was 1.195. Focusing on 4EGI-1 was unable to change the radiosensitivity of HNE1 cells under 6Gy or 8Gy exposure with increasing DR5 and PARP, this could be derived from radiation dose. It was unable to reflect the role of 4EGI-1 under large doses of radiation which caused all cells apoptosis. Also, with the increasing of dose, the more DR5 and c-PARP expression appeared.

To sum up, our work demonstrates a positive role of the death receptor-mediated apoptosis and the sensibilization to radiotherapy on inhibition of 4E-BP1 phosphorylation in the anti-tumor activities of the eIF4E/eIF4G interaction inhibitor in nasopharyngeal carcinoma. These data provide a better understanding of the potential function of 4EGI-1 and its mechanism for the cancer treatment.

## MATERIALS AND METHODS

### Cell lines

The NPC cell line 5-8F was established from fresh biopsy of NPC in Cancer Center of Sun Yat-sen University [34]. The NPC cell line HNE1 was established from fresh biopsy of NPC in Hunan Medical University [35]. The NPC cell line HK1 was established from fresh biopsy of NPC in Queen Elizabeth Hospital [36]. They all were gifted by cancer research institute, school of basic medical science, central south university (Changsha, Hunan, China). Cells were grown in RPMI 1640 (Gibco, USA) supplemented with 10% FBS (Gibco, USA) in a humidified atmosphere with 5% CO_2_ at 37°C.

### NPC and non-cancerous nasopharyngeal mucosa epithelial tissues

One hundred seventy-four paraffin-embedded NPC tissues were used for analysis of DR4 and DR5 proteins expression by immunohistochemistry (IHC). No patient had previously been treated with radiotherapy and chemotherapy at the time of original biopsy. All samples were obtained from patients before treatment at the second Xiangya Hospital of Central South University (Changsha, Hunan, China) with their informed consent. Complete clinical records and follow-up data were available for all patients. All specimens had been confirmed pathological diagnosis according to the World Health Organization histologic classification of NPC. The study was carried out after approval by the Ethics Committee of Central South University. The methods were carried out in accordance with the approved guidelines.

### IHC and scores

The IHC staining for samples on the NPC tissues and non-cancerous nasopharyngeal epithelial tissues were carried out using ready-to-use Envision TM^+^ Dual Link System-HRP methods (Dako; Carpintrria, CA). The staining condition for each antibody was adjusted according to our laboratory experience. Briefly, each section was deparaffinized and rehydrated, and high-temperature antigen retrieval was achieved by heating the samples in 0.01M citrate buffer in a domestic microwave oven at full power (750 Watts) for 30 minutes, then the samples were immersed into methanol containing 0.3% H_2_O_2_ to inactivate endogenous peroxidase at 37°C for 30 minutes. To eliminate nonspecific staining, the slides were incubated with appropriate preimmune serum for 30 minutes at room temperature. After incubation with a 1:250 dilution of primary antibody to DR5/Apo2/TRAIL-R2 (Rabbit polyclonal IgG, Catalog#: IMG-120E, Imagenex) and with a 1:500 dilution of the primary antibody to TRAIL R1/DR4 (Monoclonal IgG, Clone: B-N28, Diaclone) at 4°C overnight, slides were rinsed with phosphate-buffered saline (PBS) and incubated with a labeled polymer-HRP was added according to the manufacturer's instructions and incubated 30 minutes. Color reaction was developed by using 3, 3′-diaminobenzidine tetrachloride (DAB) chromogen solution. All slides were counterstained with hematoxylin. Positive control slides were included in every experiment in addition to the internal positive controls. The specificity of the antibody was determined with matched IgG isotype antibody as a negative control.

Positive expression of DR5 was discovered in the nucleus, a few cases also stained in cytoplasm and nucleus (Figure 1-A1 and 1-A3). Positive expression of DR4 protein was identified in cells cytoplasm, a handful of cases were positive staining both in cytoplasm and nucleus (Figure 1-B1 and 1-B3). Immunohistochemical staining of DR4 and DR5 were scored independently by JL and SF who were blinded to the clinicopathological data, at 200 x magnification light microscopy. Evaluation of staining was assessed using the intensity reactivity score (IRS) for the positive incidence of DR4 and DR5 were more than 80%, according to the literature [37], the scores standard were as followed: no staining (0), light brown (1), brown (2), and dark brown (3). DR4 and DR5 was divided into lower expression (0-1) and higher expression (2-3). Agreement between the two evaluators was 95%, and all scoring discrepancies were resolved through discussion between the two evaluators.

### Drug sensitivity assay

Cells were seeded in 96-well plates (5,000 cells per well) in 200μl complete medium and treated the next day with the agents indicated. Growth inhibition (IC50) assays were performed using the sulforhodamine B assay, as previously described [38].

### Western blotting analysis

Western blotting was carried out as previously described with a minor modification [39]. The antibodies used in the study were as followed: Rabbit polyclonal anti-DR5 antibody was purchased from ProSci, Inc (Poway, CA); mouse monoclonal anti-DR4 antibody (B-N28) was purchased from Diaclone (Stamford, CT); rabbit polyclonal anti-caspase-8, rabbit anti-poly (ADP-ribose) polymerase (PARP) and rabbit monoclonal anti-p-4EBP1 antibodies were purchased from Cell Signaling Technology, Inc (Beverly, MA); rabbit monoclonal anti-p-eIF4E (pS209) was purchased from Epitomics (USA); and rabbit polyclonal anti-GAPDH antibodies was purchased from Trevigen, Inc (Gaithersburg, MD). Quantification of signal intensity (IOD, integral optical density) was performed with Gel-Pro Analyzer software (Version 4.0). Expression change was indicated by IOD ratio of targeted protein before and after treatments. And the intensity was normalized by GAPDH signal. All detections were repeated for three independent times.

### Flow cytometric cell apoptosis detection

Apoptosis was evaluated by Annexin V staining using Annexin V-PE apoptosis detection kit purchased from BD Biosciences (San Jose, CA) following the manufacturer's instructions.

### Colony formation and radiosensitivity assays

HNE1 cells were seeded at 400 cells/well in six-well plates in triplicate and divided into 2 groups: radiotherapy group and combined 4EGI-1 treatment group. After a 24h rest, 4EGI-1 was applied in 0μM and 12.5μM according to the corresponding groups. Twenty-four hours later, after replacing the fresh complete medium, cells were treated once with radiation (0, 2, 4, 6 or 8 Gy). The colony formation assay was conducted as previously described [40].

### SiRNA-mediated gene silencing

The siRNA duplexes for nonsilencing control and DR5 were described previously [41, 42].

### Statistical analyses

Statistical analyses were performed using SPSS 19.0. The chi-square test was used to analyze the relationship between the expression of DR4 and DR5 proteins and clinicopathological characteristics of NPC. Kaplan-Meier analysis was performed for overall survival curves, and statistical significance was assessed using the log-rank test. Overall survival was defined as the time from the treatment initiation (diagnosis) to the date of death. To evaluate whether expression of DR4 and DR5 proteins were independent prognostic factors of overall survival for NPC patients, multivariate analysis using the Cox proportional hazard regression model was performed. All quantitative data are the means of three replicate determinations. Significant differences comparisons among more than two groups were performed using analysis of variance (ANOVA). All p-values were based on the two-sided statistical analysis and p-value less than 0.05 was considered to be statistically significant.
